# The short peptide encoded by long non-coding RNA RNF217-AS1 inhibits stomach cancer tumorigenesis, macrophage recruitment, and pro-inflammatory responses

**DOI:** 10.1007/s00726-024-03404-7

**Published:** 2024-07-15

**Authors:** Qi Ma, Fei Ma, Bin Zhang, Yonglei Zhang, Liangqun Peng, Xiangnan Li

**Affiliations:** 1 Department of General Surgery, Affiliated Tumor Hospital of Zhengzhou University, Zhengzhou, Henan 450000 China; 2 Department of Cerebral Surgery, The First Affiliated Hospital of Zhengzhou University, No. 1 Jianshe East Road, Erqi District, Zhengzhou, Henan 450000 China

**Keywords:** Macrophage, lncRNA, Peptide, RNF217-AS1, Stomach cancer, Inflammation

## Abstract

Certain long non-coding RNAs (lncRNAs) have potential peptide-coding abilities. Here, the role and molecular basis of the RNF217-AS1-encoded peptide in stomach cancer (SC) tumorigenesis were explored. Here, lncRNAs associated with SC pathogenesis and macrophage infiltration and lncRNAs with peptide-coding potential were searched by bioinformatics analysis. The gene mRNA and protein levels were examined by RT-qPCR and western blot assays, respectively. Cell viability, migratory, and invasive abilities were measured by CCK-8, Transwell migration, and Transwell invasion assays, respectively. The potential biological processes related to lncRNA RNF217-AS1 were identified by single-gene GSEA analysis. The effect of RNF217-AS1-encoded peptide on SC tumorigenesis was examined by mouse xenograft experiments. The results showed that lncRNA NR2F1-AS1 and RNF217-AS1 were differentially expressed and associated with macrophage infiltration in SC, and they had the ability to translate into short peptides. The RNF217-AS1 ORF-encoded peptide could reduce SC cell viability, inhibit cell migration and invasion, as well as hinder the development of SC xenograft tumors. The RNF217-AS1 ORF-encoded peptide in human SC AGS cells suppressed THP-1 cell migration, triggered the differential expression of CXCL1/CXCL2/CXCL8/CXCL12, and inactivated the TLR4/NF-κB/STAT1 signaling pathways. As a conclusion, the RNF217-AS1 ORF-encoded peptide hindered SC progression in vitro and in vivo and suppressed macrophage recruitment and pro-inflammatory responses in SC.

**Supplementary Information:**

The online version contains supplementary material available at 10.1007/s00726-024-03404-7.

## Introduction

Stomach cancer (SC), a multifactorial disease, is the fifth most frequently diagnosed cancer and the third commonest cause of cancer death worldwide (Ajani et al. 2022). SC can be induced by both genetic and environmental factors, and Helicobacter pylori infection is a well-known risk factor. Owing to the high aggressiveness with the heterogenous nature, SC is still a global problem (Machlowska et al. 2020). Stomach adenocarcinoma (STAD) is the commonest histological subtype of SC. Despite the great improvement in the management of SC over the past decades, the 5-year overall survival rate of patients with advanced disease remains low (Ajani et al. 2022). Although surgery and chemoradiotherapy can effectively control plenty of primary tumors, the conventional regimens are limited to treat these tumors at the advanced or metastatic stage. Recently, immunotherapy has emerged as an exciting and promising therapeutic option in several cancers including SC (Joshi and Badgwell 2021). However, the therapeutic efficacy of current immunologic therapies is limited and unfavorable in the treatment of cancers including SC due to the complexity of the tumor microenvironment and multitudinous unknown factors (Chang et al. 2022). Thus, it is imperative to gain an in-depth insight into the roles of immune-related cells and factors in the development of SC and the complex interaction of tumor cells, immune cells, and tumor microenvironment (Xie et al. 2021). Tumor-associated macrophages, essential members of the cancer microenvironment, are crucial for tumor development, and approaches targeting macrophages have been proposed as promising therapeutic strategies in cancer (Mantovani et al. 2022). Chemokines play vital roles in the recruitment and migration of macrophages into tumors (Mantovani et al. 2022; Propper and Balkwill 2022).

Long non-coding RNAs (lncRNAs), a class of RNA transcripts longer than 200 nucleotides in the length, are vital players in the initiation and progression of multiple cancers (Bridges et al. 2021) including SC (Lyu et al. 2022). As an example, lncRNA SNHG11 functions as an onco-lncRNA in SC by enhancing cancer cell oncogenic phenotypes by activating the β-catenin signaling (Wu et al. 2021). Conversely, lncRNA TP53TG1 regulates the ubiquitination-mediated degradation of CIP2A to suppress SC progression (Fang et al. 2022). Accumulating study shows that lncRNAs are involved in the mediation and regulation of immune responses, tumor microenvironment, and interactions of immune and cancer cells (Park et al. 2022). LINC00543 can stimulate the M2 polarization of tumor-associated macrophages and thus contributes to colorectal cancer development (Zheng et al. 2023). Moreover, various lncRNAs, such as RP11-276H19.1 and HAND2-AS1, have been shown to guide the immune microenvironment in SC patients (Wang et al. 2022).

LncRNAs have been erroneously regarded to be a group of RNA molecules with no protein/peptide-coding potential in the past (Bridges et al. 2021). However, with the advances in bioinformatics and translation omics, accumulating work discovers that some lncRNAs have protein/peptide-coding potential (Wang et al. 2019). Moreover, certain peptides encoded by lncRNAs have been demonstrated to be implicated in the tumorigenesis and progression of various cancers including SC (Chen et al. 2021). For instance, Ge et al. demonstrated that LINC00467 could encode a small peptide ATP synthase-associated peptide (ASAP), and this peptide facilitated colorectal cancer (CRC) cell proliferation in vitro and CRC xenograft tumor growth in vivo (Ge et al. 2021). Pang et al. showed that LINC00998 encoded a short peptide SMIM30, and the knockdown of SMIM30 peptide inhibited hepatocellular cancer (HCC) cell proliferation, migration, and invasion in vitro and hindered the growth and metastasis of xenograft tumors derived from HCC cells in vivo (Pang et al. 2020). Importantly, Guo and colleagues unveiled that a polypeptide CIP2A-BP generated by LINC00665 could weaken the development of triple-negative breast cancer by binding tumor oncogene CIP2A to inactivate the PI3K/AKT/NF-κB pathway (Guo et al. 2020), highlighting a novel opportunity for cancer treatment.

RNF217-AS1, an antisense transcript for RNF217, is initially deemed to be a lncRNA transcript (Fontanari Krause et al. 2014). RNF217-AS1 has been found to be differentially expressed and to be associated with the prognosis of patients in esophageal squamous cell cancer (ESCC) (Guan et al. 2020). In this study, bioinformatics, ribosome-sequencing (Ribo-seq) dataset, and mass spectrometry (MS) dataset suggested that lncRNA RNF217-AS1 had peptide-coding potential, which was also validated by western blot assay. Moreover, the effects of the RNF217-AS1-encoded small peptide on SC progression along with related molecular mechanisms were examined.

## Materials and methods

### Cell culture

Human embryonic kidney HEK293T cells, human monocyte THP-1 cells, human AGS (gastric adenocarcinoma epithelial cells, poorly differentiated) SC cells, and human HGC27 (undifferentiated) SC cells were purchased from Procell Life Science &Technology Co., Ltd. (Wuhan, China). HEK293T cells were grown in DMEM medium containing 10% FBS and 1% Penicillin-Streptomycin solution. AGS cells were cultured in Ham’s F-12 medium supplemented with 10% FBS and 1% Penicillin-Streptomycin solution. HGC27 cells were cultured in RPMI-1640 medium containing 20% FBS and 1% Penicillin-Streptomycin solution. THP-1 cells were grown in RPMI-1640 medium plus 10% FBS, 0.05 mM β-mercaptoethanol and 1% Penicillin-Streptomycin solution. All cells were maintained in a 5% CO_2_ incubator at 37˚C. All culture reagents were provided by Procell Life Science &Technology Co., Ltd.

### Plasmid construction and cell transfection

The sequences of putative ORFs of LINC01503 (ORF1), NR2F1-AS1 (ORF2), and RNF217-AS1 (ORF3) were constructed into pcDNA3.1 vector containing triple flag tags, respectively. The sequences of ORF1-3 were shown as follows: 5’-ATG CAG AGC CCT AGG TCT CCT CTC CAG AAA GAC CGG ATT TTG CCA TGT TGG CCA GGC TGG TCT-3’ for ORF1, 5’-ATG GCG GTG GCA GTG GGC CTG CAT CAC AGG TTG CAG CAG ATG TTC TCA ATA TTT CTA TTA AAA TTT CCT TAT TTC CAT ATG CAA GAG GAG CCC CAG AGC TGC ATC CTT ATG GTA GCT ACC ATG CCG-3’ for ORF2, and 5’-ATG CTT ATT AGA AAC TTA TTA GCT ACA GTA ATT TGG GGT TTC CAG TTA CAA CCA GAT GCT AGA CTA AGA TCT TTG AGG AAA AGA ACA TTG TCT CAT CTC TGC TTC TCT AAC-3’ for ORF3. Plasmids were transfected into HEK293T, AGS, and HGC27 cells using the lipo8000 transfection reagent as described by the manufacturer (Beyotime Biotechnology, Shanghai, China).

### Bioinformatics analyses and screen of lncRNAs with protein-coding potential in SC

The RNA-seq data of 26 pairs of stomach adenocarcinoma (STAD) and normal samples were obtained from the TCGA GDC database (UCSC Xena). Next, differentially expressed lncRNAs (|log2foldchange| >1 and adjusted *P*-value < 0.01) in SC versus normal samples were identified by the DEseq2 R package. LncRNAs related to macrophages in STAD (*P* < 0.05) were identified by the ImmLnc website (http://bio-bigdata.hrbmu.edu.cn/ImmLnc/). The ribosomal sequencing (Ribo-seq) dataset in HEK293T cells (GSE102720) was downloaded from the Gene Expression Omnibus (GEO) database (https://www.ncbi.nlm.nih.gov/), and the lncRNAs that could bind with ribosomes under the condition of Ribo-seq count > 0 were found out. The potential ORFs between the start and stop codons of the longest transcripts of lncRNAs were predicted by EMBOSS getorf (http://emboss.bioinformatics.nl/cgi-bin/emboss/getorf) under the condition of minimum amino acid number of 60. The mass spectrometry (MS) dataset of SC tumor tissues (PXD002674) was obtained from the ProteomeXchange website (http://www.proteomexchange.org/) and was subjected to MaxQuant processing (https://www.maxquant.org/). Single-gene gene set enrichment analysis (GSEA) analysis for lncRNA RNF217-AS1 was performed using the https://www.omicshare.com/tools/ based on the median of RNF217-AS1 expression in 26 cases of TCGA STAD samples. KEGG and GO gene sets were deemed to be significantly enriched under the conditions: NOM *p*-value < 0.05 and FDR q-value < 0.25 (Han et al. 2021).

### Western blot assay

HEK293T cells were transfected with pcDNA3.1, pcDNA3.1-ORF1, pcDNA3.1-ORF2, or pcDNA3.1-ORF3. AGS and HGC27 SC cells were transiently introduced with pcDNA3.1 or pcDNA3.1-ORF3. At 48 h after transfection, cells were collected and lysed using the RIPA lysis buffer (Beyotime Biotechnology) containing protease inhibitor cocktails (Thermo Scientific, Waltham, MA, USA). Equal amounts of protein were separated by SDS-PAGE and transferred onto nitrocellulose filter membranes (Millipore, Bedford, MA, USA). Next, the membranes were sequentially incubated with 5% skim milk for 1 h at room temperature and specific primary antibodies for about 12 h at 4˚C. Subsequently, the membranes were probed with secondary antibody conjugated with horseradish peroxidase for 1 h at room temperature. Finally, protein signals were visualized using the Pierce ECL Western Blotting Substrate (Thermo Scientific). The following primary antibodies were used: anti-flag (GB11938, 1:800, Servicebio, Wuhan, China), anti-p-p65 (ab109458, 1:3000, Abcam, Cambridge, UK), anti-p65 (ab32536, 1:10000, Abcam), anti-p-STAT1 (ab109461, 1:5000, Abcam), anti-STAT1 (ab239360, 1:1000, Abcam), anti-TLR4 (66350-1-Ig, 1:5000, Proteintech, Wuhan, China), and anti-β-actin loading buffer (81115-1-RR, 1:10000, Proteintech).

### Cell counting Kit-8 (CCK-8) assay

SC cells transfected with or without pcDNA3.1, pcDNA3.1-ORF2, or pcDNA3.1-ORF3 were seeded into 96-well plates at 1.0 × 10^3^ cells/well. At 48 h after transfection, cell viability was examined by a CCK-8 kit (Beyotime Biotechnology) according to the protocols of the manufacturer. Briefly, each well was added with 10 µl of CCK-8 solution for 1 h. Next, the absorbance was measured at 450 nm.

### Transwell migration and invasion assays

Transwell chambers (8-µm pore size, Costar Corning Inc., Corning, NY, USA) pre-coated with (for invasion) or without (for migration) Matrigel were used to examine cell invasive and migratory abilities. Briefly, cells transfected with or without pcDNA3.1, pcDNA3.1-ORF2, or pcDNA3.1-ORF3 were re-suspended in serum-free medium and seeded onto the top chambers at 5.0 × 10^4^ cells, and 600 µL of the complete medium supplemented with 10% FBS was added into the bottom part. After 24 h of incubation, cells on the upper surface were removed, and cells on the lower surface were fixed, stained with crystal violet solution (Beyotime Biotechnology), imaged, and counted.

### Mouse xenograft tumor experiments

The RNF217-AS1 ORF3 sequence was subcloned into the lentiviral expression vector pLVX-IRES-Neo to generate the recombinant plasmid pLVX-IRES-Neo-ORF3. Next, the lentiviral plasmid (pLVX-IRES-Neo (Vec) or pLVX-IRES-Neo-ORF3 (ORF3) and lentiviral packaging plasmids (psPAX2 and pMD2.G) were transfected into HEK293T cells using the lipo8000 transfection reagent. At 60 h after transfection, the media containing Vec or ORF3 lentiviral particles were collected and filtered through 0.45 μm filters. Next, AGS cells were infected with lentiviral particles. At 48 h after infection, cells were incubated with puromycin-containing media for 21 days to generate AGS cells stably transduced with Vec or ORF3 lentiviral particles.

The animal experiments were performed with the approval of the Institutional Animal Care and Use Committee of Zhengzhou University. The BALB/c nude mice (male; 6–8 weeks old) were purchased from Beijing Vital River Laboratory Animal Technology Co., Ltd. (Beijing, China). Mice were maintained in a specific-pathogen-free environment of 50-60% humidity at 21–25℃ using a 12–12 h light-dark cycle with free access to food and water. After 7 days of acclimatization, mice were randomly divided into the vector (Vec) or ORF3 group with 5 mice per group. The mice in the Vec group were subcutaneously injected with AGS cells stably transduced with Vec lentiviral particles and suspended in 100 µL of PBS (5.0 × 10^6^ cells per mouse). The mice in the ORF3 group were subcutaneously administrated with 100 µL of PBS containing AGS cells stably transduced with ORF3 lentiviral particles (5.0 × 10^6^ cells per mouse). Tumor volume was measured every 7 days using the following equation: tumor volume = (largest diameter × smallest diameter^2^)/2. All mice were sacrificed, and the xenograft tumors were resected, imaged, and weighed after 28 days.

Expression levels of PCNA, Ki67, and MMP9 in Vec or ORF3 xenograft tumors were examined by immunofluorescence (IF) assay. Briefly, resected tumor tissues were fixed and paraffin embedding. The tumor sections were blocked with 3% bovine serum albumin for 30 min and incubated for 12 h at 4˚C with primary antibody against PCNA (GB11010, 1:500, Servicebio), Ki67 (GB111499, 1:500, Servicebio), or MMP9 (GB11132-2, 1:500, Servicebio). Next, the sections were incubated for 1 h with Cy3-labelled secondary antibody (GB21303, 1:500, Servicebio) at room temperature in the dark. The cell nuclei were stained with DAPI, and the tissue autofluorescence was quenched. After mounting, the sections were imaged using fluorescent microscopy.

### RT-qPCR assay

The effect of the RNF217-AS1 ORF3-encoded short peptide on pro-inflammatory responses were evaluated by analyzing the expression change of CXCL1 and CXCL2. The isolation of total RNA from SC cells was performed using TRIzol Reagent (Thermo Scientific) according to the manufacturer’s protocols. RNA was reversely transcribed into the cDNA first strand using the RevertAid First Strand cDNA Synthesis Kit (Thermo Scientific) following the manufacturer’s instructions. Next, the quantitative PCR reaction was carried out using SYBR Select Master Mix (Thermo Scientific) and specific primers. Relative mRNA levels were calculated using the 2^−ΔΔCt^ method.

### THP-1 cell recruitment assay

AGS cells were transfected with or without pcDNA3.1 or pcDNA3.1-ORF3. At 48 h after transfection, the culture media of AGS cells transfected with or without the above-mentioned plasmids (conditioned medium) were collected and called the conditioned medium. Next, THP-1 cells resuspended in complete growth media were seeded into the upper chamber of Transwell plates (8-µm pore size, Costar Corning) at 5.0 × 10^4^ cells, and the conditioned medium was added to the lower chambers. Five hours later of incubation at 37˚C, THP-1 cells in the lower chambers were stained with Calcein AM (Servicebio), imaged, and counted.

### Statistical analysis

Data were analyzed using the GraphPad Prism software (ver. 7; La Jolla, CA, USA) and outcomes were shown as mean ± standard deviation. The difference among multiple groups was compared using the one-way ANOVA with Tukey post-hoc test or two-way ANOVA with Sidak’s multiple comparisons test. The difference between groups was analyzed using the student’s *t*-test. The difference was defined to be statistically significant when P-value was less than 0.05.

## Results

### Identification of 3 lncRNAs with potential peptide-coding ability in SC

**Fig. 1:**
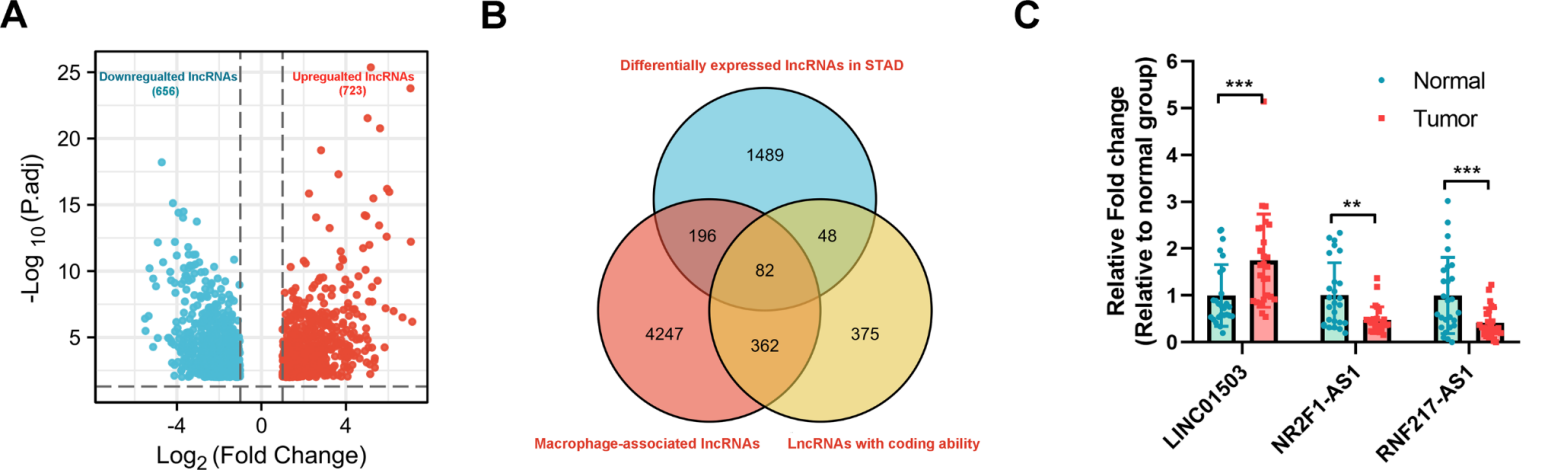
Identification of 3 lncRNAs with potential peptide-coding ability in SC. (**A**) The volcano plot of differentially expressed lncRNAs in 26 cases of SC samples than in 26 cases of paired normal samples. (**B**) Jvenn diagram of lncRNAs in lists 1–3. List 1: differentially expressed lncRNAs in TCGA STAD tumor tissues (*n* = 26) relative to paired normal tissues (*n* = 26). List 2: lncRNAs related to macrophages that were identified from the ImmLnc database. List 3: lncRNAs that could bind with ribosomes based on the GSE102720 dataset. (**C**) The histogram of LINC01503, NR2F1-AS1, and RNF217-AS1 expression profiles in 26 pairs of TCGA STAD and matching normal samples. **P < 0.01, ***P < 0.001

In this project, we downloaded the TCGA RNA-seq data of 26 pairs of STAD and corresponding normal samples. Differential expression analysis revealed that 1379 lncRNAs were differentially expressed (723 up-regulated, 656 down-regulated) in TCGA STAD samples compared with normal samples (|log2foldchange| > 1; adjusted *P-value* < 0.01) (Fig. 1A, s1 Table: list 1). These dysregulated lncRNAs might be related to the pathogenesis of SC. Given the close association between macrophages-mediated immunity and cancer progression, 4887 lncRNAs related to macrophages in STAD (*P* < 0.05) were identified by the ImmLnc website (http://bio-bigdata.hrbmu.edu.cn/ImmLnc/) (s1 Table: list 2). The common lncRNAs in lists 1 and 2 might be implicated in cancer development and macrophage-mediated immunity in SC. To further screen out lncRNAs with peptide-coding potential, ribosomal sequencing (Ribo-seq) dataset in HEK293T cells (GSE102720) rather than Ribo-seq datasets in SC samples and cells was downloaded from the Gene Expression Omnibus (GEO) database due to the deficiency of appropriate Ribo-seq datasets in SC samples and cells. Based on the GSE102720 dataset, we filtered out 867 lncRNAs that could bind with ribosomes under the condition of Ribo-seq count > 0 (s1 Table: list 3). Next, 82 common lncRNAs were identified in lists 1, 2, and 3 by jvenn analysis (Fig. 1B). We supposed that these 82 lncRNAs might be associated with macrophage-related immunity and SC development and might have the potential peptide-coding ability.

To further identify lncRNAs with peptide-coding capacity, the potential ORFs between the start and stop codons of the longest transcripts of the above-mentioned lncRNAs were predicted by the EMBOSS: getorf website (http://emboss.bioinformatics.nl/cgi-bin/emboss/getorf) under the condition of minimum amino acid number of 60. Next, we examined whether the short peptides that might be translated by the above putative ORFs could be identified in the MS dataset of SC tumor tissues (i.e. PXD002674), which was obtained from the ProteomeXchange website. MS search using the MaxQuant software showed that 8 short peptides could be identified by MS. The information about these 8 small peptides was presented in S2 Table.

Sequence comparisons using the UniProt database revealed that these 8 peptides did not match any known proteins in humans, suggesting that these peptides are uncharacterized. Among these 8 peptides-corresponding lncRNAs, 3 lncRNAs (i.e. LINC01503, NR2F1-AS1, and RNF217-AS1) were selected for further investigations because the fold-changes of these 3 lncRNAs were the highest in SC samples than in the normal samples. The differential expression profiles of LINC01503, NR2F1-AS1, and RNF217-AS1 in TCGA STAD versus paired normal samples based on the TCGA RNA-seq data were shown in Fig. 1C, analyzed by two-way ANOVA. Results revealed that LINC01503 expression was notably up-regulated and expression levels of NR2F1-AS1 and RNF217-AS1 were markedly down-regulated in TCGA STAD tumor tissues (*n* = 26) compared to the normal tissue group (*n* = 26) (Fig. 1C).

### Subcellular localization and coding-ability analysis of LINC01503, NR2F1-AS1, and RNF217-AS1

**Fig. 2:**
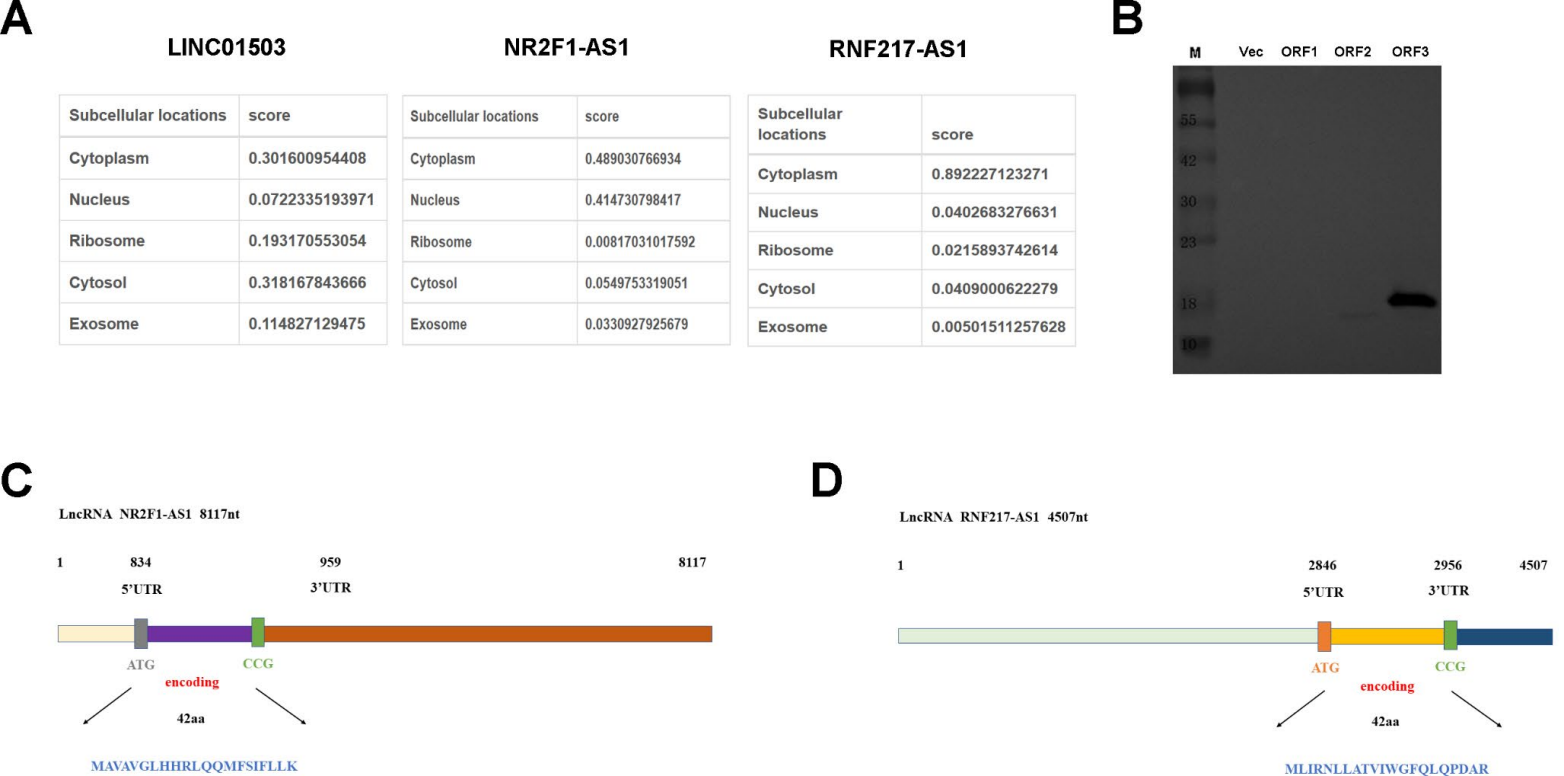
Subcellular localization and coding-ability analysis of LINC01503, NR2F1-AS1, and RNF217-AS1. (**A**) Subcellular localization patterns of LINC01503, NR2F1-AS1, and RNF217-AS1. (**B**) HEK293T cells were transfected with pcDNA3.1, pcDNA3.1-ORF1, pcDNA3.1-ORF2, pcDNA3.1-ORF3. At 48 h after transfection, the translation abilities of the above ORFs were examined by western blot assay using the antibody against the Flag tag. (**C** and **D**) The structural diagrams of NR2F1-AS1-encoded short peptide (**C**) and RNF217-AS1-encoded small peptide (**D**) on matching lncRNAs

Subcellular localization analysis by the lncLocator website (http://www.csbio.sjtu.edu.cn/bioinf/lncLocator/) suggested that LINC01503, NR2F1-AS1, and RNF217-AS1 could be found in the cytoplasm (Fig. 2A). The putative ORFs of LINC01503, NR2F1-AS1, and RNF217-AS1 were named ORF1, ORF2, and ORF3, respectively. To examine the peptide-coding abilities of ORF1, ORF2, and ORF3, these ORFs were subcloned into pcDNA3.1 vector carrying flag tag and transfected into HEK293T cells, respectively. Western blot assay using the antibody against flag tag validated that ORF2 and ORF3 could encode small peptides (Fig. 2B). The structural diagrams of the NR2F1-AS1-encoded short peptide and RNF217-AS1-encoded small peptide on corresponding lncRNAs were shown in Fig. 2C and D, respectively.

### RNF217-AS1 ORF3-derived short peptide prominently restrained SC cell viability, migration, and invasion

**Fig. 3:**
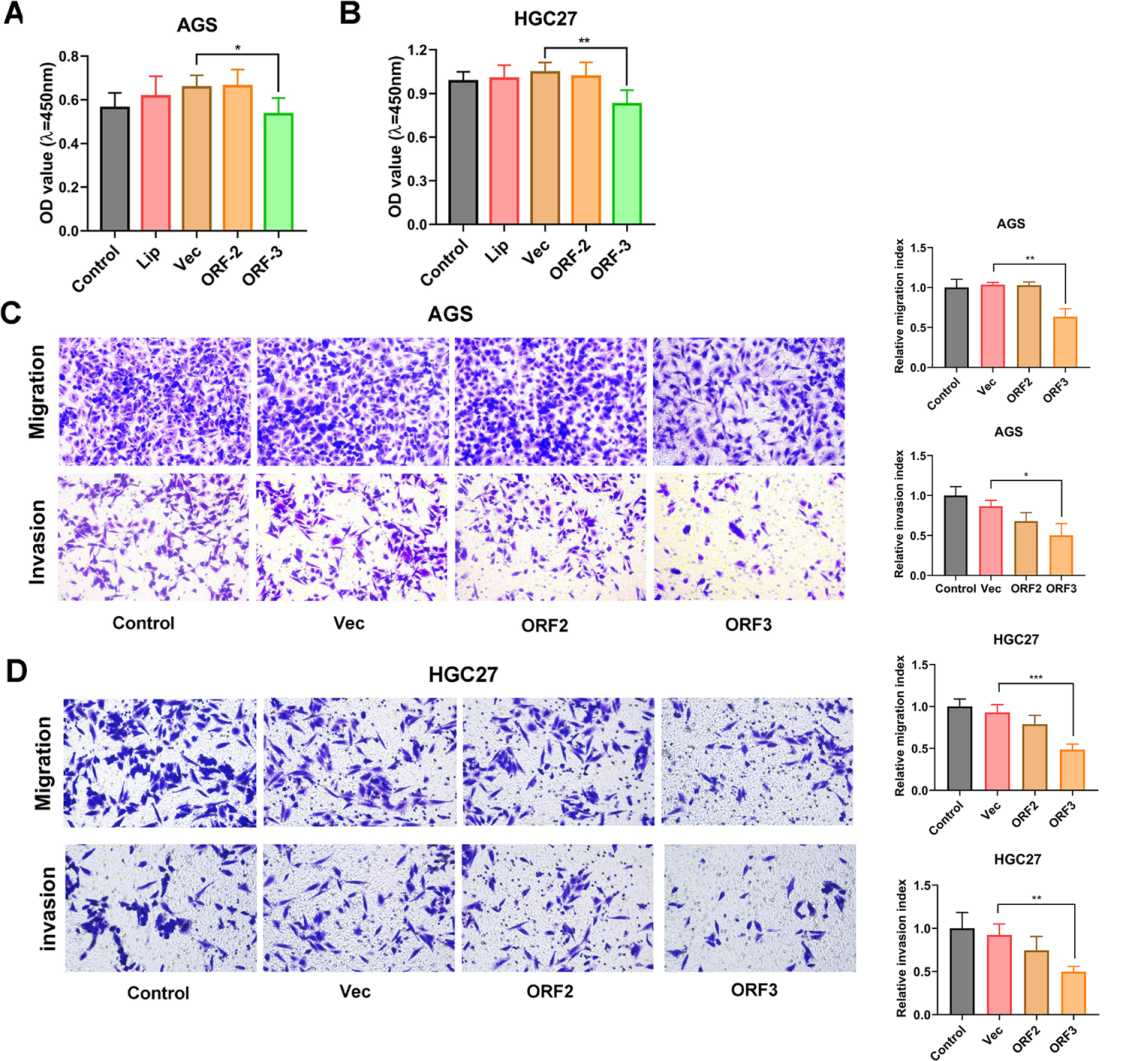
Effects of the small peptides encoded by NR2F1-AS1 ORF2 or RNF217-AS1 ORF3 on SC cell viability, migration, and invasion. (**A**-**D**) The effects of ORF2 or ORF3 overexpression on cell viability, migration, and invasion were examined by CCK-8, Transwell migration, and Transwell invasion assays in AGS and HGC27 cells, respectively. The detailed experimental procedures were shown in the part of Materials and Methods. Control: untreated cells. Lip: cells treated with lipo8000 transfection reagent. Vec: cells transfected with pcDNA3.1 empty vector. *P < 0.05, **P < 0.01, ***P < 0.001

Next, the CCK-8 assay showed that exogenous overexpression of ORF3 led to the notable reduction of cell viability in AGS (18.69% inhibition rate) and HGC27 (20.93% inhibition rate) cells (Fig. 3A and B). Transwell migration and invasion assays demonstrated that enforced expression of ORF3 markedly impaired the migratory and invasive abilities of AGS (38.85% migratory inhibition rate, 42.04% invasive inhibition rate) and HGC27 (47.80% migratory inhibition rate, 46.15% invasive inhibition rate) cells (Fig. 3C and D). We also noticed that NR2F1-AS1 ORF2 overexpression did not influence the viability, migration, and invasion of AGS and HGC27 SC cells (Fig. 3A and D). These data indicated that the RNF217-AS1 ORF3-encoded peptide could inhibit SC cell viability, migration, and invasion.

### RNF217-AS1 ORF3-encoded peptide dramatically suppressed SC xenograft tumor development in vivo

**Fig. 4:**
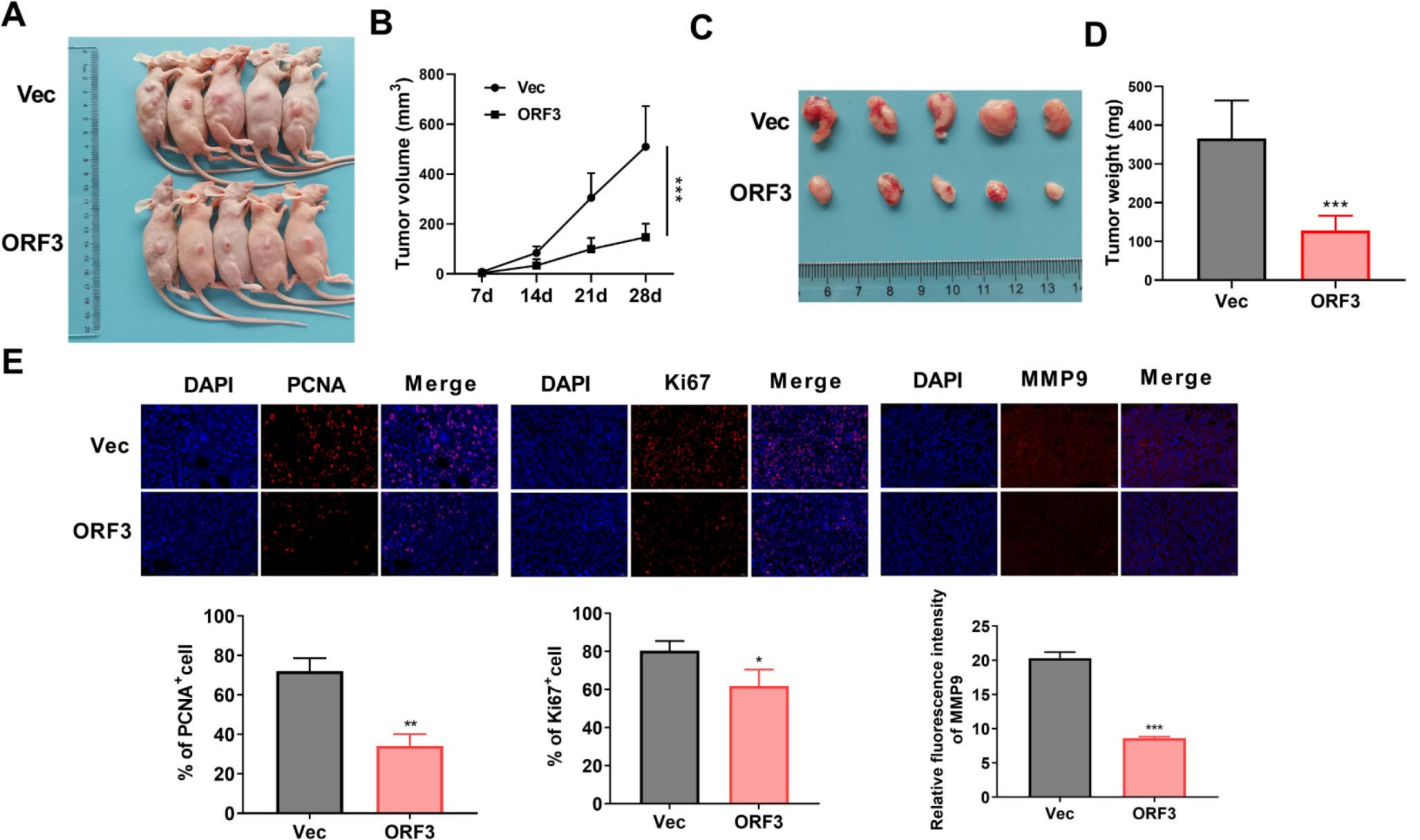
Effects of RNF217-AS1 ORF3-encoded peptide on SC xenograft tumor development. (**A**) The images of xenograft tumors in mice of vector and ORF3 groups. (**B**) The volume of SC xenograft tumors in the vector and ORF3 groups. (**C**) The images of SC xenograft tumors that were removed from mice in both vector and ORF3 groups. (**D**) The weight of removed SC xenograft tumors in the vector and ORF3 groups. (**E**) The immunofluorescence staining and intensity analysis of PCNA, Ki67, and MMP9 in the SC xenograft tumors of both vector and ORF3 groups. *P < 0.05, **P < 0.01, ***P < 0.001

Animal experiments also disclosed that RNF217-AS1 ORF3 overexpression led to a noticeable reduction in the volume (71.19% inhibition rate) and weight (65.03% inhibition rate) of SC xenograft tumors (Fig. 4A and D). Also, reduced immunofluorescence staining of PCNA (a well-known proliferation marker, 52.56% inhibition rate), Ki67 (a well-known proliferation marker, 23.07% inhibition rate), and MMP9 (a best-studied secretory endopeptidase related to tumorigenesis, 57.48% inhibition rate) was noticed in xenograft tumors stably expressing ORF3-encoded peptide compared to the vector group (Fig. 4E). Based on these data, we concluded that the RNF217-AS1 ORF3-encoded peptide could inhibit the development of SC xenograft tumors.

In addition, the expression of RNF217-AS1 ORF3-encoded peptide was determined in six pairs of stomach cancer tissues and corresponding adjacent normal tissues. The data showed that the level of RNF217-AS1 ORF3-encoded peptide was prominently reduced in cancer tissues compared with adjacent normal tissues (Sup. Figure 1).

### Single-gene GSEA analysis for lncRNA RNF217-AS1

**Fig. 5:**
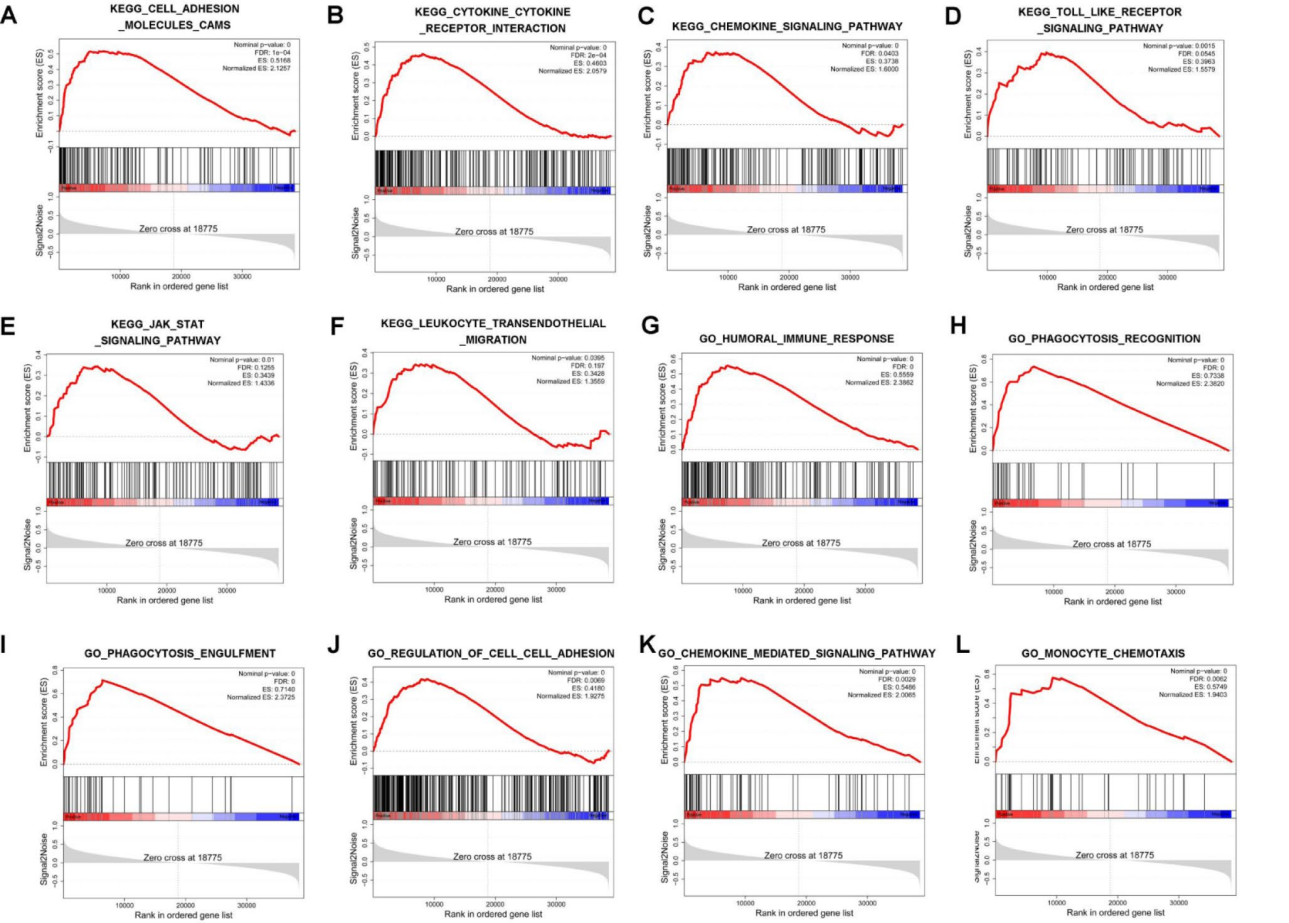
Single-gene GSEA analysis for lncRNA RNF217-AS1. (**A**-**F**) The KEGG enrichment plots of pathways such as cell adhesion molecules (**A**), cytokine-cytokine receptor interaction (**B**), chemokine signaling pathway (**C**), Toll-like receptor signaling pathway (**D**), JAK-STAT signaling pathway (**E**), and leukocyte transendothelial migration (**F**). (**G**-**L**) The GO enrichment plots of biological processes such as humoral immune response (**G**), phagocytosis recognition (**H**), phagocytosis engulfment (**I**), cell-cell adhesion (**J**), chemokine-mediated signaling pathway (**K**), and monocyte chemotaxis (**L**)

To identify potential pathways or biological processes related to ORF3-encoded short peptide, single-gene GSEA analysis for ORF3-matching lncRNA RNF217-AS1 was performed in 26 cases of TCGA STAD samples. GSEA-KEGG analysis showed that lncRNA RNF217-AS1 might play a crucial role in multiple pathways such as cell adhesion molecules, cytokine-cytokine receptor interaction, chemokine signaling pathway, Toll-like receptor signaling pathway, JAK-STAT signaling pathway, and leukocyte transendothelial migration (Fig. 5A and F, S3 Table). GSEA GO biological process analysis suggested that lncRNA RNF217-AS1 might be involved in the regulation of biological processes related to immune response, phagocytosis recognition, phagocytosis engulfment, cell-cell adhesion, chemokine-mediated signaling pathway, monocyte/macrophage chemotaxis, NF-κB import into nucleus, Toll-like receptor 4 (TLR4) signaling pathway, STAT cascade, and tyrosine phosphorylation of STAT1 protein (Fig. 5G and L, S4 Table).

### RNF217-AS1 ORF3-encoded short peptide prominently affected chemokine expression and blocked THP-1 cell recruitment

**Fig. 6:**
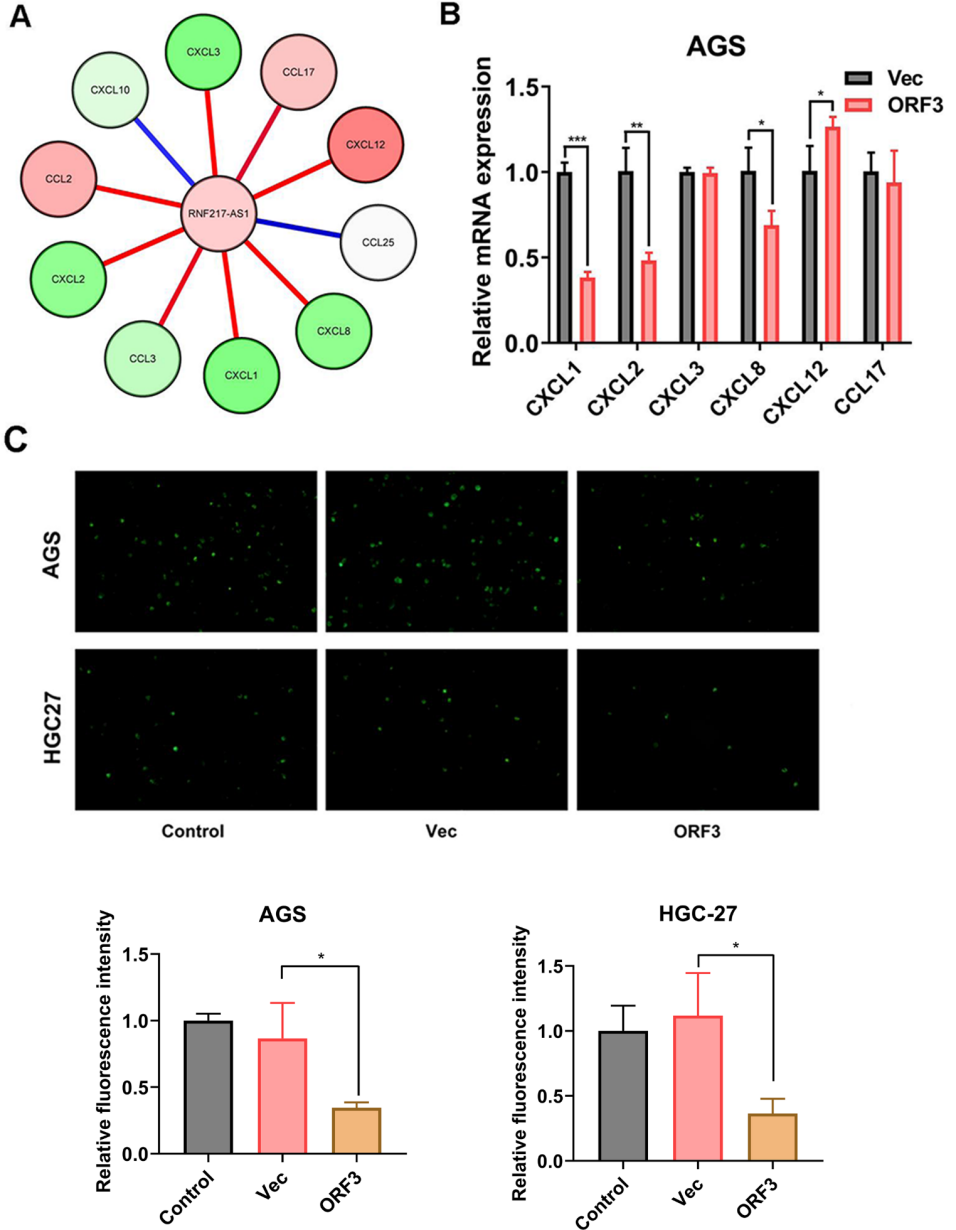
Effect of lncRNA RNF217-AS1 ORF3-encoded short peptide on chemokine expression and THP-1 cell recruitment. (**A**) The schematic diagram of associations between RNF217-AS1 and chemokines. The color of the circle denoted the correlation. Red and green circles represented positive and negative correlation relationships, respectively. The color of the line signified the significance. The red line meant that the correlation was statistically significant. The blue line indicated no statistical significance. (**B**) AGS cells were transfected with pcDNA3.1 or pcDNA3.1-ORF3. At 48 h after transfection, the mRNA levels of CXCL1, CXCL2, CXCL3, CXCL8, CXCL12, and CCL17 were measured by RT-qPCR assay. (**C**) AGS and HGC27 cells were transfected with or without pcDNA3.1 or pcDNA3.1-ORF3. At 48 h after transfection, cell supernatants (conditioned medium) were collected. The effect of the conditioned medium on THP-1 cell recruitment was examined by the THP-1 cell recruitment assay. *P < 0.05, **P < 0.01, ***P < 0.001

As mentioned above, RNF217-AS1 might play vital roles in chemokine-related signaling pathways. Thus, the associations of RNF217-AS1 and some chemokines were examined in TCGA SC samples (*n* = 373) by Pearson correlation analysis. Results showed that RNF217-AS1 expression was positively associated with the expression of CCL2, CCL17, and CXCL12, but negatively correlated with the expression of CCL3, CXCL1, CXCL2, CXCL3, and CXCL8 in 373 cases of TCGA SC samples (Fig. 6A). Moreover, RNF217-AS1 showed no association with CXCL10 or CCL25 in TCGA SC specimens (*n* = 373) (Fig. 6A). Given the close association of RNF217-AS1 and multiple chemokines, the effect of RNF217-AS1 ORF3-encoded small peptide on the expression of these chemokines was further examined by RT-qPCR assay. Results showed that the ectopic expression of RNF217-AS1 ORF3 triggered the notable reduction of CXCL1, CXCL2, and CXCL8 expression levels and a noticeable increase of CXCL12 mRNA level in AGS cells (Fig. 6B). These correlation analyses suggested that RNF217-AS1 expression was associated with the expression of multiple chemokines.

The above-mentioned GSEA analysis also suggested that RNF217-AS1 might be implicated in macrophage chemotaxis and chemokine signaling pathways. Thus, the effect of exogenous overexpression of RNF217-AS1 ORF3-encoded small peptide in SC cells on THP-1 cell migration was examined. Results showed that exogenous overexpression of ORF3 in AGS (60.12% inhibition rate) and HGC27 (67.37% inhibition rate) cells could inhibit THP-1 cell migration (Fig. 6C).

### RNF217-AS1 ORF3-encoded short peptide conspicuously inactivated TLR4/NF-κB/STAT1 signaling pathways in SC cells

**Fig. 7:**
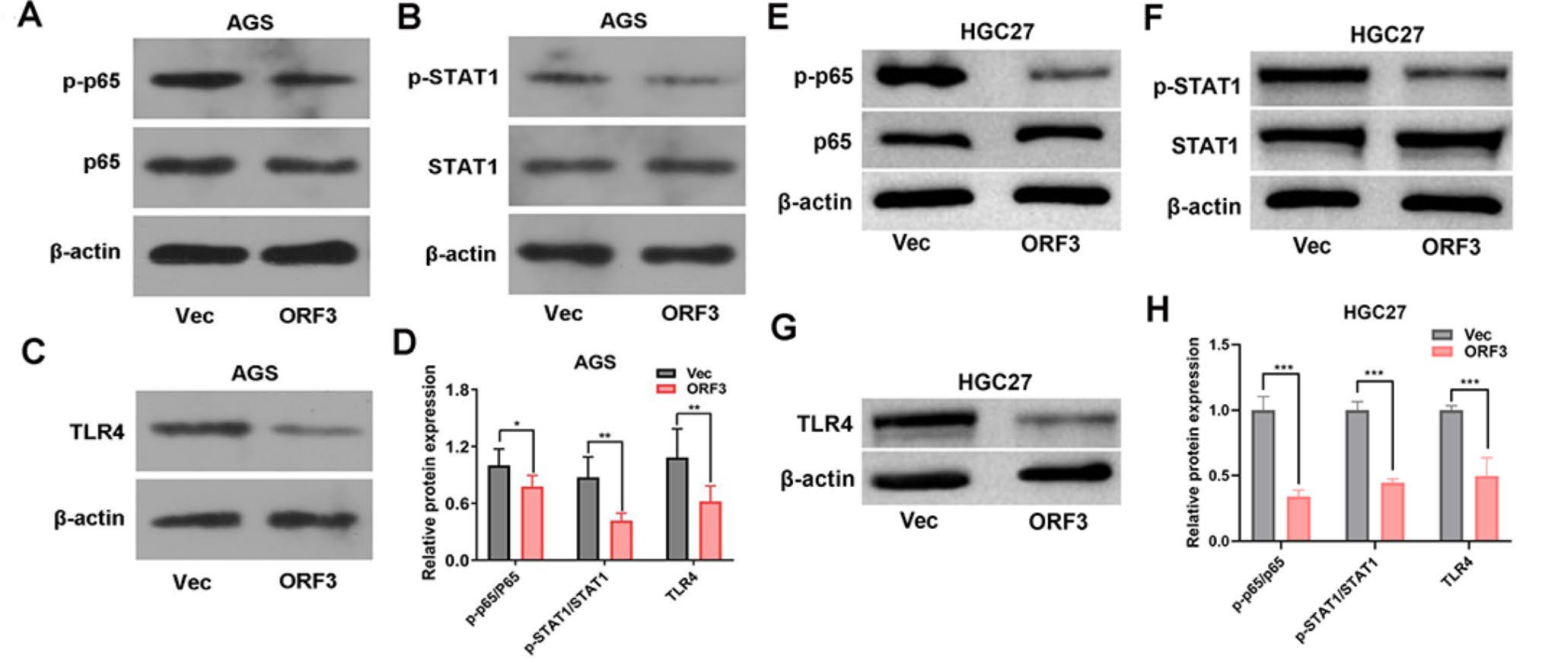
Effect of exogenous overexpression of lncRNA RNF217-AS1 ORF3-encoded short peptide on NF-κB, STAT1, and TLR4 signaling pathways in SC cells. (**A**-**H**) AGS and HGC27 cells were transfected with pcDNA3.1 or pcDNA3.1-ORF3. At 48 h after transfection, the protein levels of p-p65, p65, p-STAT1, STAT1, and TLR4 were measured by western blot assay. *P < 0.05, **P < 0.01, ***P < 0.001

Based on the GSEA analysis for the lncRNA RNF217-AS1, we supposed that the RNF217-AS1 ORF3-encoded short peptide might also be involved in the regulation of NF-κB import into nucleus, STAT1 phosphorylation, and TLR4 signaling pathway. Western blot assay further demonstrated that the exogenous overexpression of ORF3 led to the notable reduction of p-p65/p65 (AGS: 22.34% inhibition rate, HGC27: 66.01% inhibition rate) and p-STAT1/STAT1 (AGS: 52.42% inhibition rate, HGC27: 55.28% inhibition rate) ratios and TLR4 (AGS: 42.83% inhibition rate, HGC27: 50.14% inhibition rate) level in AGS and HGC27 SC cells (Fig. 7A and H), suggesting that lncRNA RNF217-AS1 ORF3-encoded short peptide could inactivate the NF-κB, STAT1, and TLR4 signaling pathways in SC cells.

Considering the tumor suppressor activity of RNF217-AS1 ORF3-encoded short peptide in SC cells, we intended to explored whether RNF217-AS1 ORF3-encoded short peptide could synergize with chemotherapy to induce SC cell death. CCK8 data showed that the ectopic expression of RNF217-AS1 ORF3-encoded short peptide significantly reduced the IC50 of 5-Fu in both SC cells (Sup. Figure 2 A and 2B), suggesting that RNF217-AS1 ORF3-encoded short peptide could sensitize SC cells to 5-Fu. These data indicated the potential of RNF217-AS1 ORF3-encoding short peptide in combination with chemotherapy in SC treatment.

## Discussion

Based on the data of bioinformatic analyses, we identified 8 dysregulated lncRNAs with peptide-coding potential and potential association with macrophage cell infiltration in SC. Among these 8 lncRNAs, we further screened out 3 lncRNAs (LINC01503, NR2F1-AS1, and RNF217-AS1) for further investigations because the fold-changes of these 3 lncRNAs were the highest in TCGA SC samples versus the normal group. LINC01503 (Ding et al. 2021), NR2F1-AS1 (Zuo et al. 2021), and RNF217-AS1 (Guan et al. 2020) have been found to be implicated in the pathogenesis of multiple cancers including SC. For instance, NR2F1-AS1 loss notably weakened the proliferative, migratory, and invasive abilities of SC cells (Li et al. 2022a, b). Miao et al. pointed out that RNF217-AS1 was differentially expressed in SC and was associated with the prognosis of SC patients (Miao et al. 2017). Thus, we further investigated whether these 3 lncRNAs could translate into small peptides and whether these lncRNA-encoded peptides were involved in the regulation of SC cell progression.

Our experiments demonstrated that the putative ORFs of NR2F1-AS1 (ORF2) and RNF217-AS1 (ORF3) could indeed translate into small peptides, while the putative ORF of LINC01503 (ORF1) could not encode a small peptide. Functional experiments demonstrated that the exogenous overexpression of the ORF3-encoded peptide markedly reduced SC cell viability, weakened SC cell migratory and invasive abilities in vitro, and notably inhibited the development of SC xenograft tumors in vivo. PCNA and MMP9 are related to cancer cell proliferation and motility, respectively, and Ki67 is a marker of cell proliferation (González-Magaña and Blanco 2020; Augoff et al. 2022). The reduced immunofluorescence staining of PCNA, Ki67, and MMP9 following this peptide expression in xenograft tumors also supported the repression of RNF217-AS1-encoded polypeptide in SC xenograft growth and metastasis in vivo.

Single-gene GSEA analysis showed that lncRNA RNF217-AS1 might be implicated in pathways or biological processes related to immunity, chemokine signaling pathway, and macrophage/monocyte chemotaxis. Chemokines and chemokine-receptor signaling have been found to be closely linked with the development and malignant progression of cancers (Korbecki et al. 2020a, b). Moreover, chemokine and chemokine-receptor signaling can reshape the phenotypes and affect the infiltration patterns of immune cells in the tumor microenvironment (Bule et al. 2021). Macrophages, an essential immune cell type in the tumor microenvironment, can be mainly divided into M1 and M2 activation phenotypes (Boutilier and Elsawa 2021). M1 macrophages mainly exhibit pro-inflammatory and anti-tumor effects, while M2 macrophages generally drive cancer progression and exert anti-inflammatory functions in cancers (Boutilier and Elsawa 2021). Previous studies have demonstrated that AGS cells-derived conditioned media induced the transformation of THP-1 cells to M2 macrophages (Zhang and Li 2020; Ngabire et al. 2020). Our present study demonstrated that exogenous overexpression of RNF217-AS1 ORF3 markedly inhibited the expression of CXCL1, CXCL2, and CXCL8 and facilitated the expression of CXCL12 in AGS cells. Moreover, exogenous overexpression of RNF217-AS1 ORF3-encoded peptide in SC cells hindered THP-1 cell recruitment. CXCL1, CXCL2, CXCL8, and CXCL12 have been found to be implicated in SC pathogenesis and macrophage recruitment and polarization (Daniel et al. 2020). For instance, CXCL1 promoted SC cell migration and SC xenograft growth, and CXCL1 expression showed a positive association with the inferior prognosis and tumor metastasis in SC (Zhou et al. 2019). CXCL2 knockdown in omental adipocytes markedly weakened the proliferative and migratory abilities of SC cells in vitro and inhibited the growth of SC xenograft tumors in vivo (Natsume et al. 2020). The release of CXCL2 activated CXCR2 and the blockade of CXCR2 signaling hindered macrophage-like cell migration that was triggered by programmed cell death 10 (PDCD10)-overexpressed glioblastoma cells (Zhang et al. 2021). The blockade of CXCL8 using the neutralizing antibody inhibited M2 polarization of macrophages induced by gastric cancer-derived mesenchymal stromal cells (GC-MSCs) and GC-MSC-primed macrophages notably improved the migratory and invasive abilities of SC cells (Li et al. 2019). We here demonstrated that exogenous overexpression of the lncRNA RNF217-AS1 ORF3-encoded short peptide led to the inactivation of NF-κB, STAT1, and TLR4 signaling pathways in SC cells. NF-κB can regulate the expression of multiple inflammatory factors including IL-8, CXCL1, and CXCL2, and the dysregulation of the NF-κB signaling pathway is closely linked with the tumorigenesis and progression of SC (Chaithongyot et al. 2021). Based on the repression of the RNF217-AS1 ORF3-encoded short peptide in the activation of the NF-κB signaling and the expression of CXCL1 and CXCL2, we concluded that this short peptide could regulate the pro-inflammatory responses in SC cells. Also, STAT1 (Li et al. 2022a, b) and TLR4 (Ito et al. 2020) signaling pathways have been found to be implicated in immune escape and tumorigenesis in SC.

Additionally, NR2F1-AS1 possesses a pro-tumorigenic effect in various cancers, including SC (Li et al. 2022a, b). Our analysis of the TCGA RNA-seq data showed the downregulation of NR2F1-AS1 in SC samples versus adjacent normal tissues, as reported by Lv et al. (Lv et al. 2021). Conversely, Zuo and colleagues demonstrated the overexpression of NR2F1-AS1 in four SC cell lines compared with normal GES-1 cells (Zuo et al. 2021). The contradictory expression pattern of NR2F1-AS1 in SC may be due to different tissue or cell types and different detection methods. The expression pattern of NR2F1-AS1 in SC tumors versus normal samples has not been identified by qPCR and may also depend on the stage of the tumors. In addition, overexpressed molecules are not necessarily carcinogenic factors. Previous work has demonstrated that polo-like kinase 1 (Plk1) is highly expressed in breast cancer, and high Plk1 expression predicts better survival in some subtypes. Although Plk1 overexpression has been found in many cancers, its upregulation has been shown to exert anti-cancer functions (Cárcer et al. 2018).

However, due to the deficiency of antibodies against RNF217-AS1-encoded small peptide, we did not measure the endogenous level of this short peptide in SC tumors and cells and did not explore the role of this small peptide in SC development at the endogenous level, which is a limitation of our study. Moreover, it is imperative to further investigate the effects of the RNF217-AS1-encoded small peptide on SC progression and macrophage infiltration in vivo. Future studies are required to generate the antibodies against RNF217-AS1-encoded small peptide to explore its endogenous expression in SC and to determine whether abnormal expression of this peptide correlates with worst patient outcome. Nonetheless, our findings provide a rationale for developing lncRNA-encoded small peptides as promising molecularly targeted therapeutic agents against SC.

## Conclusions

**Fig. 8:**
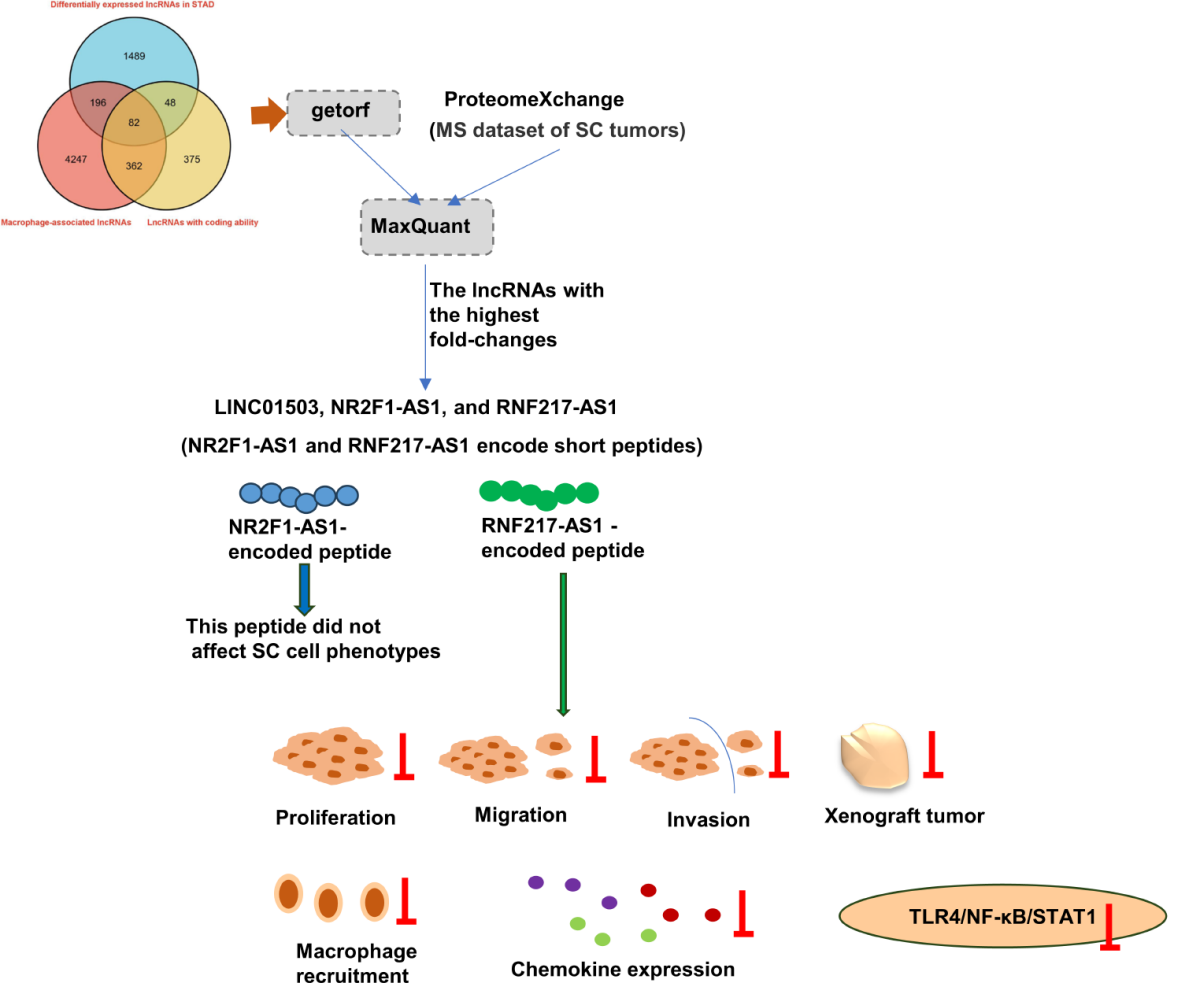
Schematic diagram of the experimental design and the main findings in this study

Taken together, our study demonstrated that lncRNAs NR2F1-AS1 and RNF217-AS1 could translate into short peptides. Functional analysis showed that RNF217-AS1-encoded short peptide could inhibit SC tumorigenesis and hinder macrophage cell recruitment in SC. This is the first study to demonstrate the peptide-coding ability of NR2F1-AS1 and RNF217-AS1 and to elucidate the vital role of the RNF217-AS1-encoded small peptide in SC development, macrophage recruitment, chemokine/TLR4/NF-κB/STAT1 signaling pathways, as illustrated in Fig. 8. Our findings could deepen the understanding of molecular mechanisms of lncRNA RNF217-AS1 and its short peptide in SC progression.

## Electronic supplementary material

Below is the link to the electronic supplementary material.

Supplementary Material 1

Supplementary Material 2

Supplementary Material 3

Supplementary Material 4

Supplementary Material 5

Supplementary Material 6

## Data Availability

No datasets were generated or analysed during the current study.
