# Supplementary figures and images for: The short peptide encoded by long non-coding RNA RNF217-AS1 inhibits stomach cancer tumorigenesis, macrophage recruitment, and pro-inflammatory responses
Source: Amino Acids. 2024 Jul 15;56(1):45. doi: 10.1007/s00726-024-03404-7 (PMC11249698; doi:10.1007/s00726-024-03404-7)

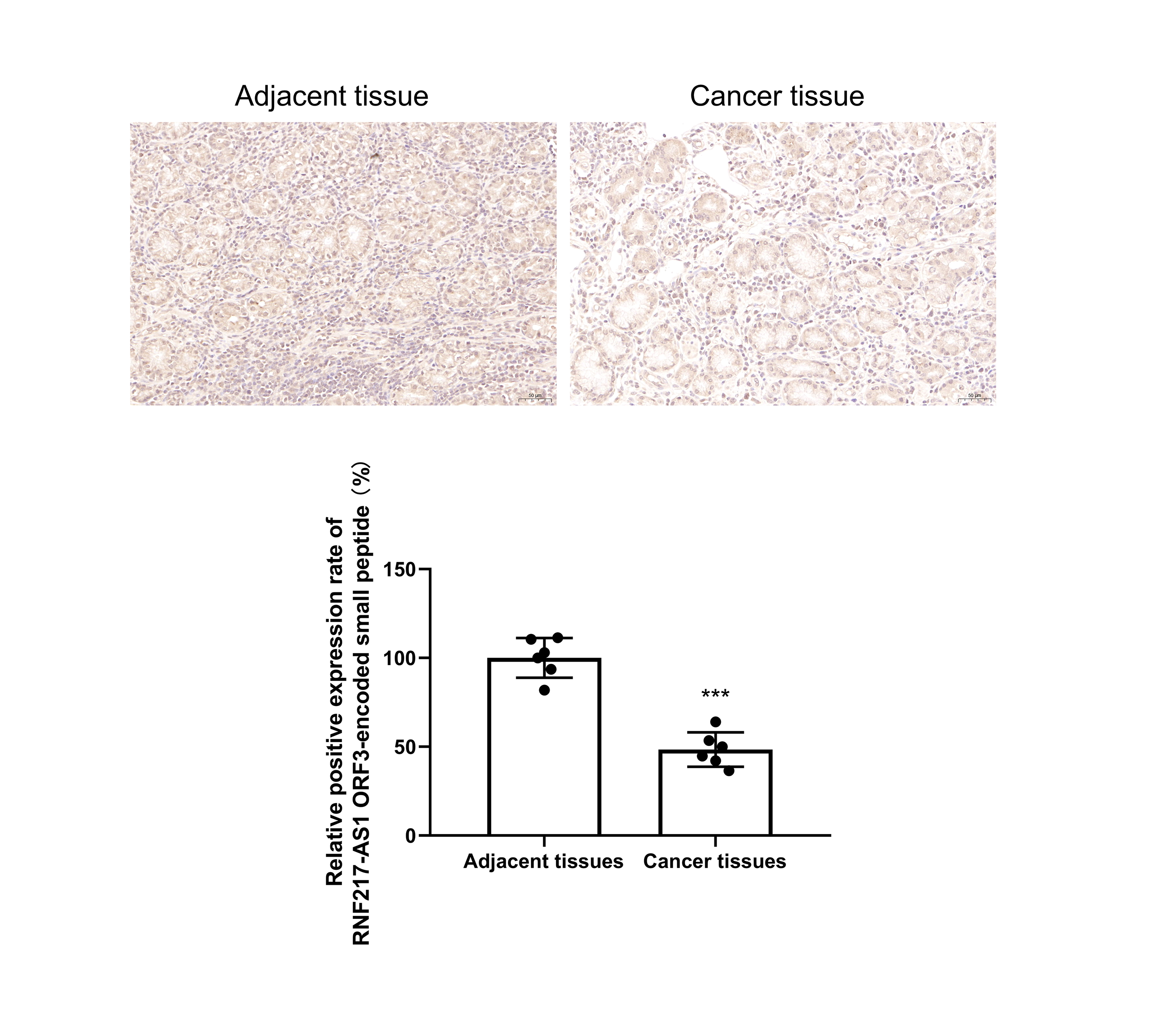

Supplement: Supplementary file 1 — Supplementary Material 1 [file 726_2024_3404_MOESM1_ESM.tif]

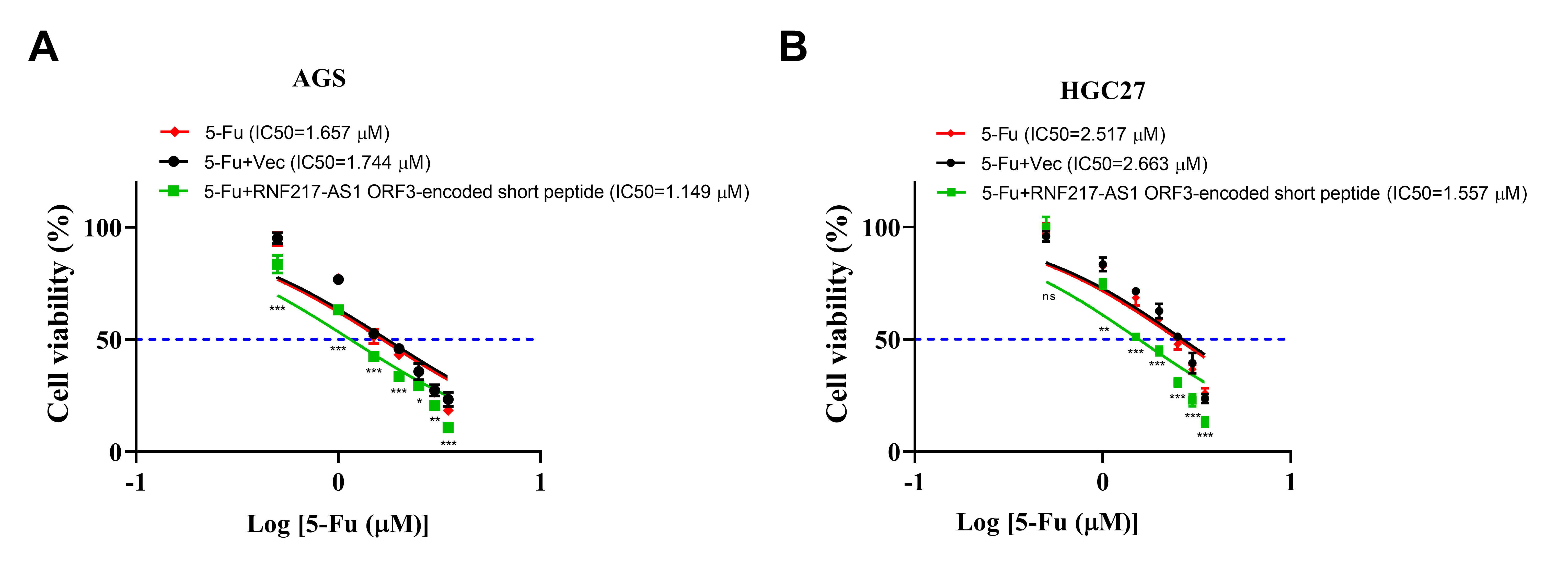

Supplement: Supplementary file 2 — Supplementary Material 2 [file 726_2024_3404_MOESM2_ESM.tif]
